# Blood Gas, Acid-Base and Electrolyte Analysis in Healthy Dromedary Camel Calves up to 21 Days of Life

**DOI:** 10.3390/ani13061117

**Published:** 2023-03-22

**Authors:** Taher Osman, Monica Probo, Davide Monaco, Hazem Karem Shafiek, Francesca Freccero

**Affiliations:** 1 Department of Advanced Biotechnology and Research, Salam Veterinary Group, Buraydah 51911, Saudi Arabia; 2 Department of Veterinary Medicine and Animal Sciences, Università degli Studi di Milano, 26900 Lodi, Italy; 3 Department of Veterinary Medicine, University of Bari Aldo Moro, 70010 Bari, Italy; 4 Department of Veterinary Medical Sciences, Università degli Studi di Bologna, 40064 Bologna, Italy

**Keywords:** acid-base, blood gas, dromedary calf, electrolytes, newborn physiology

## Abstract

**Simple Summary:**

Neonatal mortality represents a major cause of production loss in camelids, reaching rates higher than 25%; despite the increasing global importance of this species, most of the physiological features of the newborn dromedary camel remain unknown. Tailored reference values for dromedary calves are needed for a more accurate discrimination of healthy and sick animals. This study aimed at investigating the blood gases, acid-base and electrolyte profiles in healthy dromedary calves during the first 3 weeks of age, assessing possible associations with age. A total of 21 healthy dromedary camel calves aged between 1 and 21 days were sampled on the same day, and venous blood was analyzed through a VETSTAT® analyzer. Calves were divided in two groups; younger calves aged 1–10 d, and older calves aged 11–21 d. Age was associated with differences in K^+^, Na^+^, Cl^−^, sO_2_ and pCO_2_ between the two groups. These results suggest an effect of age on some blood parameters, and provide preliminary data regarding the blood gas, acid-base and electrolyte profiles in the healthy dromedary calf during the first 3 weeks of age.

**Abstract:**

The importance of prompt evaluation and care of the newborn is essential for reducing neonatal mortality, which represents a major cause of loss in camelids. This study investigated the blood gases, acid-base and electrolyte profiles in healthy dromedary calves during the first 3 weeks of life, assessing possible associations with age. Twenty-one dromedary camel calves aged 1 to 21 days were sampled, and venous whole blood analyzed through a VETSTAT® analyzer. The following parameters were measured: sodium (Na^+^), potassium (K^+^), chloride (Cl^–^), hydrogen ion concentration (pH), partial pressure carbon dioxide (pCO_2_), partial pressure oxygen (pO_2_), total hemoglobin concentration (tHb), hemoglobin oxygen saturation (sO_2_), total carbon dioxide (tCO_2_), bicarbonate (HCO_3_^–^), base excess (BE) and anion gap (AG). Calves were divided in two groups; younger calves (1–10 d), and older calves (11–21 d). Statistical analysis showed an effect of age, with lower K^+^ (*p* < 0.001) and higher Na^+^ and Cl^−^ (*p* < 0.05) mean concentrations in the younger calves compared to the older ones, and higher pCO_2_ and lower sO_2_ mean concentrations in the older group. These preliminary results firstly described the blood gas, acid-base and electrolyte profiles in the healthy dromedary calf during the first 3 weeks of age, suggesting an effect of age on some parameters.

## 1. Introduction

Old World camel species include the Dromedary (*Camelus dromedarius*) and the Bactrian (*Camelus bactrianus*), which are native to the Middle East region and Northern Africa, and to central Asia, respectively. According to a recent study [1], the world camel population is growing and should reach 60 million heads in 25 years. This growth is mainly driven by the raising demand for camel meat and milk of distinguished quality [2], and by the increased use of dromedary camels as a production animal worldwide, thanks to the high level of sustainability of camel farming and to the ability of camels to cope with heat stress [3,4,5]. The dromedary represents an important sport and tourism resource in the Arabian Gulf countries, and it is also employed for packing, transport, and riding [6,7]. In Saudi Arabia (KSA) the dromedary camel plays a primary role in the history of the Kingdom, representing a national wealth and a source of income to the majority of citizens, particularly in desert areas.

Due to seasonality, the usual calving interval for the camel is 24 months but it might easily extend to 36 months [8]; neonatal mortality, beyond that affecting animal welfare, exerts a great economic impact on farm profitability in the dromedary camel species [9]. Calf losses slow down herd’s reproduction rate, thus reducing the income for meat production. The economic importance of calf mortality is also linked to the milk production, as the presence of the calf is essential for initiation and maintenance of the lactation [8]; consequently, calf mortality threatens the sustainability of the camel farming system, and affects food and milk production in marginal areas. 

In a survey carried out in eastern Sudan, a 48% mortality rate was reported among calves under 6 months of age [10], while dromedary calf mortality rate between 30 and 50% has also been reported in Kenya [11], Tunisia [12] and Somalia [13]. In some camel herds in the UAE, dromedary calf mortality rates of up to 60% have been reported during the first 3 months of life, but with overall morbidity and mortality rates of 40% and 25%, respectively [14]. Many factors contribute to calf mortality, among which is calf diarrhea, that causes dehydration, together with electrolytes and acid-base imbalances. Inflammation of the intestinal lining impairs the calf’s ability to digest nutrients, leading to weight losses and other life-threatening disorders. A mortality rate of 39.9%, due to diarrhea, was reported in calves aged less than six months in Sudan [15]. Al-Harby et al. [16] evaluated calf scour morbidity and mortality rates in 1200 0–14 day old camel calves in Taif area (KSA); a 20% morbidity rate and 50% mortality rate were observed, respectively, with an equal distribution between winter and summer. 

Due to the critical issues highlighted above, health management of dromedary calves require care and understanding, particularly during the neonatal life, as they are susceptible to illness until they reach one year of age [8]. Notwithstanding the growing number of camels and the intensification of camel farming systems, there is a dearth in the literature regarding the physiology of newborn dromedary camels. It is essential to understand that young and adults are physiologically different, which justifies the need to obtain age-specific reference intervals. Several studies have indicated significant differences between the physiology of young and adult cattle [17,18,19,20,21], and the same can be found for other species such as horses [22], dogs [23,24] and pigs [25]. Some studies showed significant differences also in the hematological and biochemical profiles of growing dromedary calves from birth to the 4th week of age [26], and between young and adult [27,28,29]. Camel farms are often located far from diagnostic facilities and laboratories, and assessment of sick dromedary calves is still commonly mainly based on physical examination [30,31,32]. However, Old World camelids are legendary in their ability to preserve water, and this adaptation may hamper the accurate identification of early hypovolemia in critically ill camelids by physical examination alone, making the clinical estimation of fluid deficits particularly challenging [33]; for example, their small, ovoid erythrocytes are resistant to osmotic changes, and can continue to circulate in situations of increased blood viscosity. The increasing availability of pen-side blood gas analyzers can facilitate diagnostic work-up and permit evaluation of degree and nature of alterations in acid-base and electrolyte balance, which usually accompany diarrhea and other neonatal diseases. Blood gas and electrolyte analysis combined with appropriate reference ranges for healthy animals represents a prompt and useful tool in the early detection and close monitoring of disturbances; early diagnosis and treatment of neonatal disease, particularly infections, result in greater positive outcome [34]. Mean values for blood gas analysis of newborn dromedary calves immediately after birth have been investigated [26], but no information is available regarding the blood gas and acid-base profile in dromedary calves during the first weeks after birth. Tailored reference values for dromedary camel calves are urgently needed for more accurate differentiation of healthy and sick animals. 

Therefore, the aim of the present study was to investigate the blood gas, acid-base and electrolyte profiles of healthy dromedary calves up to 21 days of life.

## 2. Materials and Methods

### 2.1. Sample Population

In March 2022, twenty-one dromedary camel calves aged between 1 and 21 days (d) were sampled. The animals were reared through a semi-intensive breeding system in a farm located in the Al-Qassim region (Centre of Saudi Arabia). About one hundred dromedary camel females were reared on the farm, distributed into pregnant and calf-rearing dams. The paddocks were provided with shaded areas. Pregnant animals were left free to graze on pastures for three hours/day and received alfa-alfa upon their return. Calf-rearing animals received alfa-alfa and concentrates. For both groups water was provided at libitum. All procedures were performed on the same day, to avoid environmental biases. The health status of each calf was assessed on the day of sampling through complete physical examination performed by veterinarians: body temperature, calf demeanor, mobility and reactivity, suckle reflex, feed intake, fecal consistency and hydration status were evaluated. Only calves that were deemed clinically healthy were enrolled in the study. 

### 2.2. Blood Sampling and Analysis

Each calf was blood sampled by jugular venipuncture. A venous blood sample was collected from the jugular vein into 2.5 mL lithium-heparin syringes, and immediately analyzed by a VETSTAT® analyzer (IDEXX, Westbrook, ME, USA), with individual body temperature set for each calf. Before testing, all visible air bubbles were expelled from the syringe, which was continuously agitated to prevent formation of microclots. Single-use disposable cassettes were employed for the assessment of sodium (Na^+^, mmol/L), potassium (K^+^, mmol/L), chloride (Cl^–^, mmol/L), hydrogen ion concentration (pH), partial pressure carbon dioxide (pCO_2_, mmHg), partial pressure oxygen (pO_2_, mmHg), total hemoglobin concentration (tHb, g/dL), hemoglobin oxygen saturation (sO_2_, %), total carbon dioxide (tCO_2_, mmol/L), bicarbonate (HCO_3_^–^, mmol/L), base excess (BE, mmol/L) and anion gap (AG, mmol/L) in whole blood. Before performing the analysis, a temperature-correction was applied for each sample on the basis of the rectal temperature registered during clinical examination of the subject. To ensure optimal performance, standard reference cassette (SRC) measurements were checked on the day of sampling, by using SRC level 1 and SRC level 3 provided by the manufacturer.

### 2.3. Data Collection and Statistical Analysis

Calves were divided into 2 groups according to their age: between 1 and 10 d of age (*n* = 10), and between 11 and 21 d of age (*n* = 11). Mean values (±standard deviation, SD) for each parameter were calculated for both groups, and statistical analysis was applied to determine differences between the two age groups for each parameter (Student t-test for independent samples). Subsequently, the linear regression analysis was performed on the total number of calves to assess the possible effect and magnitude of each day of increasing age on the studied parameters. Statistical analysis was performed using Jamovi® ver. 2.3.21 for Windows. Significance was set for *p* < 0.05. 

## 3. Results

Among the 21 calves, 12 were males and 9 were females. Mean age for the 1–10 d group was 4.3 d, while it was 15.4 d for the 11–21 d group. Statistic for the pooled data is shown in Table 1. Regression analysis showed that the age of the calf was associated with changes in some of the variables evaluated. Specifically, younger neonates (1–10 d) had significantly lower mean K^+^ concentrations (*p* < 0.001), and higher mean Na^+^ (*p* < 0.05) and Cl^−^ concentrations (*p* < 0.05) compared to older calves (11–21 d). Regarding blood gas values, pCO_2_ was lower for younger calves (*p* < 0.05) while sO_2_ was higher (*p* < 0.05) compared to 11–21 d old calves.

Linear regression analysis showed that the age of the calf, independently by the groups, was associated with changes in some of the variables evaluated, with diverse magnitude. In fact, K^+^ was shown to increase of 0.06 mmol/L each day of increasing age (*p* < 0.001); Cl^−^ decrease of 0.26 mmol/L each day of increasing age (*p* < 0.05); sO_2_ decrease of 0.58% each day of increasing age (*p* < 0.05) and pCO_2_ showed a tendency to increase of 0.47 mmHg (*p* = 0.05). Mean concentrations of Na^+^ decreased significantly from younger to older dromedary calves (*p* < 0.05) but not linearly.

## 4. Discussion

In view of the increasing interest for the dromedary species and due to the paucity of knowledge regarding laboratory parameters, particularly when different ages are considered, this study focused the attention on physiological age-specific values of blood gases, electrolytes and acid-base in healthy dromedary calves up to 3 weeks of life. In the assessment of age as an independent variable, a single cut-off point of 10 d was applied, in view of previous findings on newborn bovine calves, which detected most significant changes in blood gases and electrolytes profile before and after 10 days of age [35]. The point-of-care (POC) analyzer employed in the present study was not specifically validated for camelids, but a recent study demonstrated that a similar POC analyzer can be a valid blood gas and biochemistry instrument for use with venous blood from clinically healthy New World camelids [36].

The hypothesis that reference ranges for blood gas and electrolyte parameters should be specific for the age of the patient was proven in other species [35,37,38,39], and corroborated also in the dromedary species by the results of the present study. The age of the dromedary calves has been in fact significantly associated with differences in both blood gas analysis and electrolyte. Being the first study reporting blood gas, acid-base and electrolytes concentrations in the young dromedary calf, the comparison with previous results in age-matched dromedaries is unfeasible, while some discussion can be made in comparison with newborn dromedaries immediately after birth [26], with adult dromedaries [40], and with newborns of other species. 

Electrolytes concentrations were affected by the age of the calves. Mean concentrations of K^+^, Na^+^ and Cl^−^ registered in 1–10 days old calves in the current study were similar to the mean concentrations reported by Tharwat et al. [26] in dromedary calves immediately after birth (4.4 ± 1 mmol/L, 156 ± 2.2 mmol/L and 116 ± 1 mmol/L, respectively). The significant rise of blood concentrations of K^+^ and the decrease of blood Cl^−^ and Na^+^ concentrations from 1–10 d old calves to 11–21 d old calves in the present study seem to suggest a still on-going maturation process of homeostasis in the newborn. A study on donkey foals also showed an increase in K^+^ concentrations from samples collected within 1 day after birth to samples collected at day 14 and 21 of life [39], while no significant changes were found in Na^+^ and Cl^−^ concentrations with age [39]. A study on equine foals showed no differences in the whole electrolyte profile during the first year of life [41], and therefore proposed combined average mean values for the electrolyte profile in foals aged <1 year. On the contrary, Viesselmann et al. [36] found that the age of the calf influenced the electrolyte profile in the bovine species, but with decreasing K^+^ and increasing Na^+^ and Cl^−^ concentrations from 1–10 d old calves to 11–30 d old calves, in contrast with the present findings on the dromedary calf. Age-related differences in electrolytes concentrations have been reported for llamas [42]; reference ranges reported for Na^+^, K^+^ and Cl^−^ in adult llamas (148–156 mmol/L, 4–5.3 mmol/L and 110–124 mmol/L, respectively) and in adult alpacas (140–156 mmol/L, 4–5.8 mmol/L and 104–119 mmol/L, respectively) were very similar to those found in the present study in the older dromedary calves group (11–21 d). This finding suggests that electrolytes concentrations in healthy calves older than 10 days of age are already comparable to those of adults [40].

Blood gas analysis was performed on venous blood, and although some parameters related to the oxygenation ability are affected by the sampling site, some others were reported to be similar in arterial and venous blood in humans [43,44,45]. No correlation has been shown between arterial and venous blood samples for pO_2_ and sO_2_ in some animal species, underlining that reliable measurement for pulmonary gas exchange evaluation should only be performed on arterial blood samples [46,47]. Moreover, camelids have small, ellipsoid red blood cells with an unusually high concentration of hemoglobin, and this factor can affect the accuracy of oximetry in camelids [48]. For new world camelids (namely llama and alpaca), an arterial sample is recommended for the evaluation of respiratory parameters, preferably paired with a venous sample [49], as arterial parameters are influenced by of the high hemoglobin affinity for oxygen in these species. This implies that the present findings regarding sO_2_ on dromedary calves cannot reflect the effective percentage of hemoglobin fully combined with oxygen, lacking in clinical significance. Instead, studies in dogs [50,51] and equine [52,53] suggested that venous pH, pCO_2_ and actual HCO_3_^-^ measurements are in sufficient agreement with the arterial value, provided that circulatory status is not impaired. Arterial sampling can be technically difficult [54,55] particularly in hypovolemic subjects, and carries the risk for some complications, especially when dealing with large animals under field conditions. The jugular vein can provide representative samples of whole-body metabolism, since this vessel is of large caliber and has normally relatively high blood flow [56]. Therefore, the choice for sampling venous blood in this research was made in order to obtain an accurate but also easily replicable method to assess blood gas, acid-base and electrolyte values in young dromedary calves, especially to support diagnosis in critical emergency situations. As the influence of body temperature on the values of some blood gas parameters has been previously demonstrated [46], a temperature-correction was applied for each sample analysis on the basis of the rectal temperature registered during the clinical examination; this is particularly important considering that pH, pO_2_ and pCO_2_ values are automatically corrected according to the patient temperature by the VETSTAT® analyzer.

Mean pCO_2_ concentrations were affected by age in the present study, with increasing values in older dromedary calves; this could be partly ascribed to differences in respiratory rate according to age. The pCO_2_ values in 1–10 d old dromedary calves (43–57 mmHg) were similar to those reported for age-matched newborn bovine calves (43–58 mmHg) [35], but higher than those reported for newborn foals (42.2 ±2.1 mmHg) and donkey foals (37.1–38.9, [39]; 42.9–41.2, [47]). In these last studies however, researchers sampled the newborns immediately after birth and until 96 hours after birth, thus involving younger newborns compared to those of the present study. The high pCO_2_ values registered in the older dromedary calves group (11–21 d) in this study were similar to those from adult dromedaries [40], suggesting that high pCO_2_ can be a distinctive feature of the old world camelids, maybe an attribute adaptive for life in areas with high environmental temperatures and low water availability, and that this feature is already expressed in calves older than 10 days of age. Concentrations of tCO_2_ were not different in the two groups in the present study, and they were in agreement with those reported in the adult dromedary [40].

No difference in pH values were found in the present study between the different age groups, underlining how lungs, kidneys, and the buffer system interact and respond to physiologic changes in order to tightly regulate blood pH [57]. The mean pH values of both groups in this study (7.34–7.38) were higher than the mean pH registered in the newborn dromedary calf at birth (7.29 ± 0.03) [26], and superimposable to the mean pH reported for adult dromedary (7.35 ± 0.03) [40]. When compared to newborns of other species, the dromedary calves show the lowest pH range values (7.26–7.4); pH ranges were in fact 7.37–7.5 in healthy newborn calves within 30 days after birth [35], 7.35–7.5 in the juvenile llama <1 year [49], and 7.41–7.48 in the donkey foals at 24 hours of age [39]. The fact that a similar pH range was registered in the adult dromedary (7.28–7.44) [40], suggests a distinctive trait of this species, and needs to be taken into account when a clinical evaluation of sick dromedary calves is performed, in order to avoid overestimation of acidemia or underestimation of alkalemia in this species. While a minimum pH cut-off value of 7.36 is suggested for diagnosing acidemia in bovine calves aged 7 to 26 d [58], the present study suggests that a pH value of 7.34–7.38 can be considered as normal for healthy dromedary calves between 1–21 d of age, as these values were found to be the mean pH values in the two groups of healthy dromedary calves enrolled. 

None of the other acid-base parameters significantly differed among calves of different ages. This may be due to the fact that, within few days after birth, the main metabolic adaptations to the extrauterine life have already taken place; when evaluating hematological and biochemical parameters in the newborn of many species according to age, in fact, most of the significant changes were detected during the very first hours after birth [47,59,60], mostly due to the effect of parturition and to the newborn adaptation to the extrauterine life. Regarding HCO_3_^−^, the mean and lower and upper limits registered in the present study were very similar to those reported in the adult dromedary [40] and in the newborn donkey foal [39,47], while lower and higher mean HCO_3_^−^ concentrations were reported in 7-day-old foals [59] and in the calf [35], respectively. The BE, commonly used in evaluating the metabolic status of patients [61] ranged from −1.1 to 4.3, almost superimposable to the range reported for adult dromedaries (−2 to 4) [40]; when compared to newborns of other species, mean BE concentrations in the present study were lower than those reported in healthy bovine calves of similar ages [35], and more similar to findings on the newborn foals and donkey foals [47,56]. 

The linear regression analysis showed that a progressive increase of K^+^, decrease of Cl^−^ and sO_2_, and borderline increase of pCO_2_ is seen when all the calves were considered as a whole unique group, highlighting that these changes occur linearly, along age progression.

In the present study, some efforts were adopted to avoid possible confounding variables; a single farm was enrolled, and all dromedary calves were sampled on the same day, in order to reduce farm and environmental influences on blood results. All these aspects reduced the availability of calves that could be enrolled, representing a limitation of the study. Due to the special environmental conditions in which dromedaries are reared, and to their unique physiology especially regarding water metabolism, specific blood variable ranges are needed for this species, and according to the present study, age should be considered as an influencing factor. 

## 5. Conclusions

Although the number of calves enrolled prevents from establishing definite reference ranges, the present study provides preliminary data for evaluating blood gas, acid-base and electrolyte concentrations in young dromedary calves. This is of fundamental importance in the diagnosis and correction of imbalances resulting from the most common diseases affecting the newborns during the first month of life, such as diarrhea. According to the present study, when compared to newborns of other species, the dromedary calf presents some unique characteristics, such as a lower pH range and higher pCO_2_ concentrations. Further studies on a greater number of calves are advisable to corroborate these results, and to evaluate possible influences of other variables such as gender, breed and ease of parturition on the blood gas, acid-base and electrolyte profile of the dromedary calf.

## Figures and Tables

**Table 1:** Mean (±SD) data and min-max ranges for each venous blood gas analysis variable in the two age-groups of clinically healthy dromedary calves.

Variable	Group	N	Mean	SD	Range
Temperature (°C)	1–10 d	10	38.5	0.28	38.1–39
	11–21 d	11	38.4	0.26	38–38.7
Na^+^ (mmol/L)	1–10 d	10	154.4 *	4.30	147–163
	11–21 d	11	151.2	2.71	147–155
K^+^ (mmol/L)	1–10 d	10	4.0 **	0.44	3.2–4.6
	11–21 d	11	4.9	0.44	4.3–5.6
Cl^−^ (mmol/L)	1–10 d	10	116.9 *	4.01	111–124
	11–21 d	11	114.1	2.12	111–117
pH	1–10 d	10	7.38	0.02	7.35–7.4
	11–21 d	11	7.34	0.05	7.26–7.4
pCO_2_ (mmHg)	1–10 d	10	50.3 *	4.22	43–57
	11–21 d	11	57.5	6.98	50–71
pO_2_ (mmHg)	1–10 d	10	37.6	16.14	24–37
	11–21 d	11	31.9	2.74	27–36
sO_2_ (%)	1–10 d	10	66.3 *	8.18	62–80
	11–21 d	11	58.8	4.77	60–67
tCO_2_ (mmol/L)	1–10 d	10	28.6	2.10	24.3–31
	11–21 d	11	30.60	1.41	28.1–33
BE (mmol/L)	1–10 d	10	2.1	1.61	−1.1–4.3
	11–21 d	11	2.0	1.65	0.0–4.2
HCO_3_^-^ (mmol/L)	1–10 d	10	27.1	1.99	23.0–29.3
	11–21 d	11	28.4	1.32	26.5–31
AG (mmol/L)	1–10 d	10	14.2	1.32	12.8–16.6
	11–21 d	11	13.7	1.78	9.7–15.9
tHb (g/dL)	1–10 d	10	11.3	2.12	5.4–12.8
	11–21 d	11	11.1	2.01	7.5–14.4

* Significant differences between groups with *p* < 0.05; ** Significant differences between groups with *p* < 0.001.

## Data Availability

The data presented in this study are available on request from the corresponding author.

## References

[1] Faye B. (2020). How many large camelids in the world? A synthetic analysis of the world camel demographic changes. Pastoralism.

[2] Wernery U., Kaaden O.R. (1995). Infectious Diseases of Camelids.

[3] Hoffmann I. (2010). Climate change and the characterization, breeding and conservation of animal genetic resources. Anim. Genet..

[4] El-Harrak M., Martín-Folgar R., Llorente F., Fernández-Pacheco P., Brun A., Figuerola J., Jiménez-Clavero M.A. (2011). Rift Valley and West Nile virus antibodies in camels, North Africa. Emerg. Infect. Dis..

[5] Wako G., Tadesse M., Angassa A. (2017). Camel management as an adaptive strategy to climate change by pastoralists in southern Ethiopia. Ecol. Process..

[6] Wilson R.T. (1984). The Camel.

[7] Faye B. (2016). The camel, new challenges for a sustainable development. Trop. Anim. Health. Prod..

[8] Manefield G.W., Tinson A.H. (1997). Camels A Compendium.

[9] Salih O.M., Shigidi H.O., Mohamed Y. The bacterial causes of camel-calf diarrhea in eastern Sudan. Proceedings of the Third Annual Meeting for Animal Production Under Arid Conditions.

[10] Agab H., Abbas B. (1999). Epidemiological studies on camel diseases in eastern Sudan. World Anim. Rev..

[11] Mukasa-Mugerwa E. (1981). The Camel (Camelus dromedarius): A Bibliographical Review; International Livestock Centre for Africa (ILCA).

[12] Burgemeister R. (1974). Problems of Dromedary Management and Breeding in Southern Tunisia. Ph.D. Thesis.

[13] Hussein M.A. (1987). Traditional Practices of Camel Husbandry and Management in Somalia.

[14] Tibary A., Anouassi A. Obstetrics and neonatal cares. Theriogenology Camelidae, Anatomy, Physiology, Pathology and Artificial Breeding.

[15] Ali Y.H., Khalafalla A.I., Gaffar M.E. (2005). Group A rotavirus-associated camel calf diarrhea in Sudan. J. Anim. Vet. Adv..

[16] Al-Harby M.S. (2013). The prevalence of new born calf-camel scours with special reflection to epidemiology; bacterial etiology and physiology processing at taif, KSA. Assiut Vet. Med. J..

[17] Adams L.G., Polzin D.J. (1989). Mixed acid-base disorders. Vet. Clin. North Am. Small Anim. Pract..

[18] Gustin P., Detry B., Robert A., Cao M.L., Lessire F., Cambier C., Katz V., Ansay M., Frans A., Clerbaux T. (1997). Influence of age and breed on the binding of oxygen to red blood cells of bovine calves. J. Appl. Physiol..

[19] Lorenz I., Gentile A., Klee W. (2005). Investigations of d-lactate metabolism and the clinical signs of d-lactataemia in calves. Vet. Rec..

[20] Mohri M., Sharifi K., Eidi S. (2007). Hematology and serum biochemistry of Holstein dairy calves: Age related changes and comparison with blood composition in adults. Res. Vet. Sci..

[21] Koch A., Kaske M. (2008). Clinical efficacy of intravenous hypertonic saline solution or hypertonic bicarbonate solution in the treatment of inappetent calves with neonatal diarrhea. J. Vet. Intern. Med..

[22] Faramarzi B., Rich L. (2019). Haematological profile in foals during the first year of life. Vet Rec..

[23] Kuhl S., Mischke R., Lund C., Gunzel-Apel A.R. (2000). Reference values of chemical blood parameters in puppies during the first eight weeks of life. Deut. Tierarztl Woch..

[24] Lund C., Kuhl S., Mischke R., Gunzel-Apel A.R. (2000). Reference values of the red blood profile in beagle, German shepherd and golden retriever puppies. Berl. Munch. Tierarztl Wochenschr..

[25] Ventrella D., Dondi F., Barone F., Serafini F., Elmi A., Giunti M., Romagnoli N., Forni M., Bacci M.L. (2017). The biomedical piglet: Establishing reference intervals for haematology and clinical chemistry parameters of two age groups with and without iron supplementation. BMC Vet. Res..

[26] Tharwat M. (2015). Haematology, biochemistry and blood gas analysis in healthy female dromedary camels, their calves and umbilical cord blood at spontaneous parturition. J. Camel Pract. Res..

[27] Rezakhani A., Habibabadi S.N., Ghojogh M.M. (1997). Studies on normal haematological and biochemical parameters of Turkmen camel in Iran. J. Camel Pract. Res..

[28] Al-Busadah K.A., Osman T.E.A. (2000). Haematological Parameters of Adult Dry, Lactating and Camel Calves in Saudi Arabia. Pak. J. Biol. Sci..

[29] Gaashan M.M., Al-Mubarak A.I.A., Hussen J. (2020). Leukocyte populations and their cell adhesion molecules expression in newborn dromedary camel calves. Vet. World.

[30] Schwartz H.J., Dioli M. (1992). The One-Humped Camel in Eastern Africa. A Pictorial Guide to Diseases, Health Care and Management.

[31] Basheir B.O., ElMalik K.H., Abdelgadir A.E., Gameel A.A.R. (2012). Traditional and modern practices in the diagnosis, treatment and prevention of animal diseases in South Kordofan State; Sudan. J. Cell Anim. Biol..

[32] Volpato G., Lamin Saleh S.M., Di Nardo A. (2015). Ethnoveterinary of Sahrawi pastoralists of Western Sahara: Camel diseases and remedies. J. Ethnobiol. Ethnomed..

[33] Bedenice D. (2009). Approach to the Critically Ill Camelid. Vet. Clin. Food. Anim..

[34] Dolente B.A., Lindborg S., Palmer J.E., Wilkins P.A. (2007). Culture-positive sepsis in neonatal camelids: 21 cases. J. Vet. Intern. Med..

[35] Dillane P., Lea Krump L., Aideen Kennedy A., Sayers R.G., Sayers G.P. (2018). Establishing blood gas ranges in healthy bovine neonates differentiated by age, sex, and breed type. J. Dairy Sci..

[36] Viesselmann L.C., Videla R., Flatland B. (2018). Verification of the Heska Element Point-of-Care blood gas instrument for use with venous blood from alpacas and llamas, with determination of reference intervals. Vet. Clin. Pathol..

[37] Meyer D.J., Harvey J.W. (2004). Veterinary Laboratory Medicine: Interpretation & Diagnosis.

[38] Roland L., Drillich M., Iwersen M. (2014). Hematology as a diagnostic tool in bovine medicine. J. Vet. Diagn. Investig..

[39] Veronesi M.C., Gloria A., Panzani S., Sfirro M.P., Carluccio A., Contri A. (2014). Blood analysis in newborn donkeys: Hematology, biochemistry, and blood gases analysis. Theriogenology.

[40] Abdoun K.A., Samara E.M., Okab A.B., Al-Haidary A.A. (2012). State of Acid-base Balance in Dehydrated Camels (*Camelus dromedarius*). Asian. J. Anim. Vet. Adv..

[41] Bauer J.E., Harvey J.W., Asquith R.L. (1984). Clinical chemistry reference values of foals during the first year of life. Eq. Vet. J..

[42] Fowler M.E., Zinkl J.G. (1989). Reference ranges for hematologic and serum biochemical values in llamas (*Lama glama*). Am. J. Vet. Res..

[43] Moore E.E., Good J.T. (1982). Mixed venous and arterial pH: A comparison during hemorrhagic shock and hypothermia. Ann. Emerg. Med..

[44] Kelly A.M., McAlpine R., Kyle E. (2001). Venous pH can safely replace arterial pH in the initial evaluation of patients in the emergency department. Emergency Med. J..

[45] Middleton P., Kelly A., Brown J., Robertson M. (2006). Agreement between arterial and central venous values for pH, bicarbonate; base excess, and lactate. Emergency Med. J..

[46] Bleul U., Lejeune B., Schwantag S., Kahn W. (2007). Blood gas and acid-base analysis of arterial blood in 57 newborn calves. Vet. Rec..

[47] Carluccio A., Contri A., Gloria A., Veronesi M.C., Sfirro M.P., Parrillo S., Robbe D. (2017). Correlation between some arterial and venous blood gas parameters in healthy newborn Martina Franca donkey foals from birth to 96 hours of age. Theriogenology.

[48] Grubb T.L., Anderson D.E. (2017). Assessment of clinical application of pulse oximetry probes in llamas and alpacas. Vet. Med. Sci..

[49] Garry F. (1989). Clinical Pathology of Llamas. Vet. Clin. N. Am. Food Anim. Pract..

[50] Van Sluijs F.J., De Vries H.W., De Bruijne J.J., Van den Brom W.E. (1983). Capillary and venous blood compared with arterial blood in the measurement of acid-base and blood gas status of dogs. Am. J. Vet. Res..

[51] Ilkiw J.E., Rose R.J., Martin I.C. (1991). A comparison of simultaneously collected arterial, mixed venous, jugular venous and cephalic venous blood samples in the assessment of blood-gas and acid–base status in the dog. J. Vet. Internal Med..

[52] Speirs C. (1980). Arteriovenous and arteriocentral venous relationships for pH, PCO2, and actual bicarbonate in equine blood samples. Am. J. Vet. Res..

[53] Hauser B., Wehrend A., Bostedt H., Failing K. (2001). The diagnostic value of venous blood gas parameters and pH value in newborn foals with pulmonary diseases. Berl. Munch. Tierarztl. Wochenschr..

[54] Fisher E.W., Sibartie D., Grimshaw W.T.R. (1980). A comparison of the pH, pCO_2_, pO_2_ and total CO_2_ content in blood from the brachial and caudal arteries in normal cattle. Br. Vet. J..

[55] Verhoeff J., Wierda A. (1983). Use of blood from arterialized capillaries in the ears of calves for the analysis of pO_2_, pCO_2_, pH and bicarbonate. Res. Vet. Sci..

[56] Hodgson D.R. (1987). Blood Gas and Acid-Base Changes in the Neonatal Foal. Vet. Clin. N. Am. Eq..

[57] Palmer J., Langdon Fielding C., Gary Magdesian K. (2015). Acid–base homeostasis and derangements. Equine Fluid Therapy.

[58] Sayers R.G., Kennedy A., Krump L., Sayers G.P., Kennedy E. (2016). An observational study using blood gas analysis to assess neonatal calf diarrhea and subsequent recovery with a European Commission-compliant oral electrolyte solution. J. Dairy Sci..

[59] Kitchen H., Rossdale P.D. (1975). Metabolic profiles of newborn foals. J. Reprod. Fertil. Suppl..

[60] Probo M., Giordano A., Moretti P., Opsomer G., Fiems L., Paltrinieri S., Veronesi M.C. (2019). Serum biochemical profile in Holstein Friesian and Belgian blue calves in the first 48 hours of life. Ital. J. Anim. Sci..

[61] Constable P.D. (2000). Clinical assessment of acid-base status: Comparison of the Henderson-Hasselbalch and strong ion approaches. Vet. Clin. Pathol..

